# A giant spleen with multiple cysts: a rare case of isolated splenic hemangiomatosis

**DOI:** 10.11604/pamj.2021.39.42.29712

**Published:** 2021-05-17

**Authors:** Tapan Patel, Shivani Patel

**Affiliations:** 1 Department of General Surgery, Baroda Medical College, Vadodara, India

**Keywords:** Splenic hemangiomatosis, giant spleen, isolated hemangiomatosis

## Image in medicine

A 63-year-old female presented with the complaint of lump in left upper quadrant of abdomen since two years which had gradually increased in size reaching up to umbilicus. It was associated with dull aching abdominal pain. Her vitals and initial laboratory investigations were in normal range. Her family history was insignificant. Contrast enhanced computed tomography (CECT) of abdomen revealed a grossly enlarged spleen with parenchyma greatly replaced by cysts of variable sizes, largest one measuring 72mm x 69mm x 69mm. Few cysts had peripheral discontinuous rim like calcifications (A,B). Splenectomy done via subcostal incision revealed a spleen weighing 1800 gram and measuring 28cm x 16cm x 9cm with irregular surface showing numerous cysts filled with serous and serosanguinous fluid (C,D). Normal architecture of spleen was replaced almost completely by cysts filled with proteinaceous fluid containing red blood cells. There was dystrophic calcification in the periphery of the cysts. The endothelial cells were diffusely positive for CD34 and factor VIII, focally positive for CD31 but not for CD8. The diagnosis of splenic hemangiomatosis was made. Splenic hemangiomatosis is extremely rare with unknown incidence and is most frequently seen in cases of diffuse angiomatosis. The imaging characteristics of splenic hemangiomas are non-specific. The differential for a cystic lesion of the spleen includes lymphoma, metastases, abscess, cyst, lymphangioma and hematoma. Due to poor specificity of imaging and the risks associated with percutaneous biopsy of the spleen, splenectomy is performed when definitive characterization of splenic lesions is needed.

**Figure 1:**
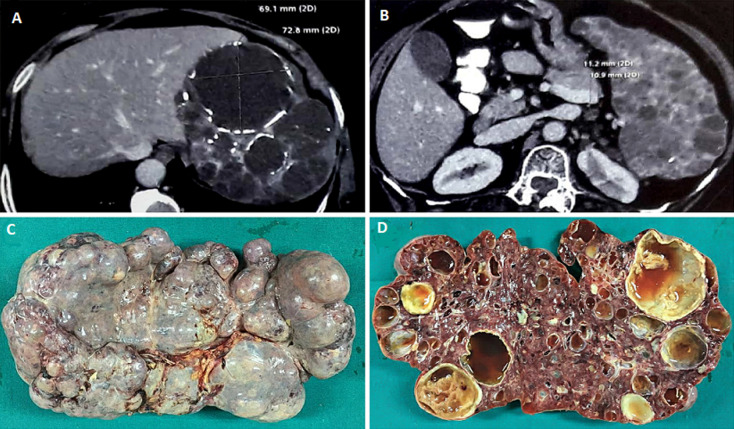
A) CECT abdomen suggestive of spleen with multiple cysts, few of them are having incomplete rim like calcifications; B) CECT abdomen suggestive of massive splenomegaly with normal appearing right kidney; C) gross specimen of a massive spleen with irregular surface; D) Cut section of spleen suggestive of normal spleen texture almost completely replaced by numerous cysts containing serous or serosanguinous fluid

